# Global hepatitis elimination 2030: immediate action is the need of the hour

**DOI:** 10.1016/j.eclinm.2024.102763

**Published:** 2024-07-23

**Authors:** eClinicalMedicine

The disease we know today as viral hepatitis, can be traced back to 5th century Greece as epidemics of jaundice, along with multiple documented outbreaks in the 19th and 20th centuries. Hepatitis is caused by a group of viruses: hepatitis A, B, C, D, and E. Hepatitis A and E are spread from infected food and water, whereas hepatitis B, C, and D are spread by infected blood and bodily fluids. Hepatitis A and E usually cause self-limiting disease, but the bulk of global health issues are due to hepatitis B and C. These two viruses can cause chronic hepatitis and are the leading cause of hepatocellular carcinoma. Viral hepatitis is the second leading viral cause of death globally after COVID-19.

Viral hepatitis is more prevalent in low and middle-income countries (LMICs), which account for 80% of the global hepatitis burden. Of these, nine countries (Bangladesh, China, Ethiopia, India, Indonesia, Nigeria, Pakistan, Philippines and Vietnam) and the Russian Federation are responsible for two-thirds of this burden. In April 2024, WHO released its first consolidated report on viral hepatitis, at the World Hepatitis Summit in Lisbon, Portugal. An estimated 254 million people are living with hepatitis B and 50 million are living with hepatitis C globally. In 2022, 2.2 million people were newly infected by viral hepatitis, of which, 1.2 million were hepatitis B infections and nearly 1.0 million were hepatitis C infections. The report also estimated that the number of deaths from viral hepatitis increased from 1.1 million in 2019 to 1.3 million in 2022, caused mainly by hepatitis B (83%) and hepatitis C (17%).

Specific populations are at a higher risk of infection, including people with a history of exposure with unsafe needles, frequent blood transfusions and improper sterilisation during procedures. Newborns and children are especially at risk through vertical (mother-to-child) transmission of hepatitis B. Most of the global burden of chronic hepatitis B can be attributed to vertical transmission of hepatitis B virus peripartum or through horizontal transmission in early childhood.

Despite having established treatment and prevention strategies for viral hepatitis, we are off track for the WHO goal for global elimination by 2030. This is majorly because the global coverage for viral hepatitis prevention, diagnosis, and treatment is low. Unfortunately, by the end of 2022, a mere 13% of people living with chronic hepatitis B infection had been diagnosed, and an even smaller proportion (3%) received antiviral therapy. Additionally, only 36% of people living with hepatitis C were diagnosed between 2015 and 2022, and only 20% had received curative treatment.

July 28th is World Hepatitis Day, and this year the theme ‘Take Action. Test, Treat and Vaccinate’ pushes us to factor in effective change in all three domains, if the world intends to meet the 2030 deadline. The vaccine against hepatitis B virus has been around since 1969, and now, more than 50 years later, 115 countries have made monovalent hepatitis B vaccination within 24 h of birth, followed by additional two to three doses in early childhood; a part of their immunisation schedules. Of the 38 WHO focus countries for the viral hepatitis response, 25 countries have introduced birth-dose programmes, and all provide the hepatitis B vaccine (usually as part of pentavalent vaccines) in their infant vaccination programme. However, coverage varies, and is reported to be as low as 18% in the WHO African region. The monovalent hepatitis B virus is also available for at risk adults (health-care workers, patients receiving frequent transfusions, etc) in a three-dose schedule. Unfortunately, we are yet to have a vaccine against hepatitis C, which would be an important milestone in this struggle.

There are several testing methods for viral hepatitis like rapid diagnostic tests and immune assays, however the testing remains largely centralised and there is little use at community grass-root levels. Although, countries have developed national plans and adopted WHO testing guidelines, the implementation of these policies remains low, and there is no targeted funding and manpower required to meet these testing needs.

Despite prices of generic hepatitis medications being low, many countries cannot access these drugs at these prices because of policy, poor accessibility, and corruption. The prices of these generic medications lack uniformity and in many countries, especially in the African region, affected populations need to pay out of their own pocket, which is a huge constraint in LMICs. Although tenofovir disoproxil for hepatitis B is the national guidelines of 94% of the WHO focus countries, it is only available in 45% at a primary health-care level. Similarly, sofosbuvir and daclatasvir or ledipasvir for hepatitis C are only available in 30% of these counties at a primary health-care level. Additionally, only 40% of these countries have registered hepatitis B medication for children.

With only 6 years left until the 2030 deadline, we need to put our collective efforts into overcoming these remaining challenges. WHO has recommended several focal points for 2024–26 to achieve the desired results to be back on track for 2030. These include delivering people-centric services such as quality affordable testing, optimising systems through product regulation, increasing financing for health service delivery, generating health data and using these data to drive decisive actions, engaging communities for self-advocacy, and fostering innovations including a hepatitis B cure and hepatitis C vaccine. The time has come for us to wake up and take collective action for a healthier tomorrow.

